# The role of socioeconomic and climatic factors in the spatio-temporal variation of human rabies in China

**DOI:** 10.1186/s12879-018-3427-8

**Published:** 2018-10-22

**Authors:** Danhuai Guo, Wenwu Yin, Hongjie Yu, Jean-Claude Thill, Weishi Yang, Feng Chen, Deqiang Wang

**Affiliations:** 1 Computer Network Information Center, Chinese Academy of Sciences, 4th South Fourth Road Zhongguancun, Beijing, 100190 China; 2 University of Chinese Academy of Sciences, 19th Yuquan Road, Beijing, 100049 China; 3 Chinese Center for Disease Control and Prevention, 155 Changbai Road Changping District, Beijing, 102206 China; 4 Department of Geography & Earth Sciences, The University of North Carolina at Charlotte, 9201 University City Blvd, Charlotte, NC 28223 USA; 5 School of Geography and Planning, Sun Yat-sen University, Guangzhou, 510275 China; 6 Key Laboratory of Land Surface Pattern and Simulation, Institute of Geographic Sciences and Natural Resources Research, Chinese Academy of Sciences, Beijing, 100101 China; 7 Department of East Asian Studies, The University of Arizona, 1512 E. First Street, Tucson, AZ 85719 USA

**Keywords:** Human rabies, Socioeconomic and climate factors, Regression model, Heterogeneity, Spatial dependence, China

## Abstract

**Background:**

Rabies is a significant public health problem in China. Previous spatial epidemiological studies have helped understand the epidemiology of animal and human rabies in China. However, quantification of effects derived from relevant factors was insufficient and complex spatial interactions were not well articulated, which may lead to non-negligible bias. In this study, we aimed to quantify the role of socio-economic and climate factors in the spatial distribution of human rabies to support decision making pertaining to rabies control in China.

**Methods:**

We conducted a multivariate analysis of human rabies in China with explicit consideration for spatial heterogeneity and spatial dependence effects. The panel of 20,368 cases reported between 2005 and 2013 and their socio-economic and climate factors was implemented in regression models. Several significant covariates were extracted, including the longitude, the average temperature, the distance to county center, the distance to the road network and the distance to the nearest rabies case. The GMM was adopted to provide unbiased estimation with respect to heterogeneity and spatial autocorrelation.

**Results:**

The analysis explained the inferred relationships between the counts of cases aggregated to 271 spatially-defined cells and the explanatory variables. The results suggested that temperature, longitude, the distance to county centers and the distance to the road network are positively associated with the local incidence of human rabies while the distance to newly occurred rabies cases has a negative correlation. With heterogeneity and spatial autocorrelation taken into consideration, the estimation of regression models performed better.

**Conclusions:**

It was found that climatic and socioeconomic factors have significant influence on the spread of human rabies in China as they continuously affect the living environments of humans and animals, which critically impacts on how timely local citizens can gain access to post-exposure prophylactic services. Moreover, through comparisons between traditional regression models and the aggregation model that allows for heterogeneity and spatial effects, we demonstrated the validity and advantage of the aggregation model. It outperformed the existing models and decreased the estimation bias brought by omission of the spatial heterogeneity and spatial dependence effects. Statistical results are readily translated into public health policy takeaways.

## Background

Rabies is a widely distributed zoonotic infectious disease. The latest estimates indicate that a total of 55,000 human fatalities occur each year worldwide as a result of rabies infection [1–3]. After India, China is the country with the second highest annual incidence of human rabies cases, where humans contract the infection from rabid animals. The rabies virus mainly spreads from animal to animal or animal to human through bites and scratches [4]. Among reservoirs animals, dogs play a pivotal role as a transmitter of rabies to humans in China [5] and it is estimated that more than four fifths of all human rabies infections in China are due to dogs [4]. Although the number of cases has decreased over the past decade, the epidemic situation remains serious and numerous cases have been reported in recent years: 924 cases in 2014 and 801 cases in 2015 [3, 6]. Unsuccessful control of rabid animals and inadequate post-exposure prophylaxis (PEP) of patients are thought to be the main factors leading to the high incidence of human rabies in China [7, 8].

Multiple studies have dug into the epidemiology and transmission dynamics of rabies to humans in China across various temporal and geographic scales [4, 7–14]. Phylogenetic analysis of Chinese rabies viruses from 1969 to 2009 illustrated that due to human-related activities infection transmission had been intra-provincial and extra-provincial [9]. Time-series analysis of human rabies has shown seasonal trends: the number of cases in summer and autumn is higher than in spring and winter [4, 12, 15]. In the only spatial epidemiological study of rabies in dogs, it can be seen that the spatial and temporal distribution of cases is not even across the country [16]. Cases of rabies infection in areas where there was no prior history of infection are reported yearly [16]. Investigation of spatial patterns of rabies in animals such as raccoons and skunks have shown obvious variations because of the diversity in geographic, climatic and environmental attributes: distance from major roads, presence of river, lake and land cover including deciduous forest, average temperature and nearness to enzootic zones have all been found to be covariates [17–19]. More recently, surveillance data have been exploited in spatial models to forecast the emergence of rabies in raccoons [20, 21]. However, the impact of risk factors on observed spatial distributions has so far not been studied quantitatively.

The quantification of risk factors associated with the occurrence of human rabies is critical for the epidemiologic analysis of rabies, as this knowledge crucially supports decision making for controlling and ultimately preventing the disease. A significant goal of the analysis is to predict the incidence of rabies or possibilities of rabies cases for specific regions. In order to predict feasibly and reliably, quantification of the risk factors is vital. A number of international studies have investigated the contribution of risk factors in the spatial distribution of rabies in humans [20, 22–24]. Some studies have looked for a correlation between rabies exposure risk and socioeconomic status using measurements derived from the records of patient PEP [25, 26]. However, these studies did not consider the spatio-temporal variation in environmental factors. The heterogeneity of local regions was also neglected. In addition, variations derived from spatially lagged geographical variables was inadequately accounted for in these models. Similarly designed studies have also been conducted for other infectious diseases [27–29].

In China, the spatial analysis of human rabies has shown that, while the overall number of rabies cases in humans decreased from 2007 to 2011, the scope of infection is still expanding [4]. It has been reported by numerous studies that the transmission of rabies was not restricted by administrative boundaries and the history of occurrence of the disease, but was impacted by surrounding environments, economic conditions and human habits, which all exhibit spatial heterogeneity [7, 30]. However, it remains unclear what the socioeconomic and environmental cause of human rabies are.

It is necessary to analyze rabies risk at the case level in order to accurately identify the influence of the geographic environment and of socioeconomic determinants on rabies distribution. The spatial pattern of human rabies has strong relations with the distribution and movement of animals like untied dogs and roaming dogs [24]. In this paper, we analyze the space-time distribution of rabies among humans, and conduct a large-scale study based on cases of human rabies.

Besides the possibilities of risk factors mentioned above, spatial heterogeneity and spatial dependence are also important issues to account for. Spatial heterogeneity refers to the variability of environmental and social factors across a study region, while spatial dependence refers to attributes of a spatial entity or region being correlated to attributes of another nearby entity, and vice versa. Few studies of human rabies have so far explicitly considered these effects. Heterogeneity is present and considerable, especially for a large country comprised of numerous regions that may exhibit totally different natural environments and socioeconomic complexions from one another. Heterogeneity is the result of the complicated aggregation of local factors, which may not be noted accurately or completely recognized, and thus may be overlooked in the inferential analysis. In addition, when the spatial arrangement of regions is not neutral and when their neighborhood relations interfere with epidemiologic processes, an effective analysis has to consider the spatial autocorrelation in the process under study, which reveals the interactions and interdependencies between events and attributes based on their geographic proximity. A biased estimation of effects of risk factors can result from the omission of neighborhood-based spatial dependence effects.

In this study, we aim to quantify the role of socioeconomic and environmental factors of human rabies risk in China. To this end, various regression models are used to capture the relations between normalized annual counts of rabies cases and relevant explanatory variables, which are extracted from the quantitative measurement of risk factors. To account for spatial heterogeneity and spatial dependence, panel data that encompass cross-sectional information and longitudinal variation are needed for the regression estimation. A spatially autoregressive error process is integrated to handle the spatial dependence effects [31]. Spatial heterogeneity can be handled through the specification of the error component. Then, seemingly unrelated regression (SUR) estimators are used for the computation of effects on the panel data. The estimation of effects from risk factors is expected to contribute to the design of better interventions in the context of public health decision making, which is aimed at reducing cases of rabies.

In Section “Methods”, the theoretical model and econometric specifications are introduced. Section “Modeling Results” describes the data for the regression and relevant processing. Estimation results of different regression models are represented in Section “Discussion”. Section “Conclusions” provides more detailed discussions of the estimation results in relation to the epidemiology of rabies infection. The last section summarizes the analysis and presents our conclusions.

## Methods

Our analysis aims to build a model that provides explanation of the count of rabies cases and quantifies the respective effects of explanatory variables. The count of cases is used to present the degree of the incidence in geographically referenced territories across time. The model is built on panel data, which can output views through the spatial dimension and the temporal dimension. As mentioned above, we incorporate spatial heterogeneity and spatial dependence effects into the model. Before designing an appropriate model, the presence of spatial autocorrelation in the distribution of rabies cases is validated through Moran’s I statistic. When the spatial autocorrelation is determined to be significant, we start with a reduced form of rabies regression and proceed to extend the model with the heterogeneity and spatial correlations taken into consideration. Moreover, the incidence may be influenced by unobserved variables and random factors. Therefore, a random effects model is adopted in the following analysis.

### Moran’s I statistic

In order to test for the presence of spatial autocorrelation, Moran’s I statistic [32] is implemented. Moran’s I is widely applied in evaluating spatial autocorrelation in univariate areal data series. The index is defined as:

$$ Mora{n}^{\prime }s\ I=\frac{\sum_{i=1}^n{\sum}_{j=1}^n{W}_{ij}\left({y}_i-\overline{y}\right)\left({y}_j-\overline{y}\right)}{S^2{\sum}_{i=1}^n{\sum}_{j=1}^n{W}_{ij}} $$

$$ {S}^2=\frac{\sum_{i=1}^n{\left({y}_i-\overline{y}\right)}^2}{n},\overline{y}=\frac{\sum_{i=1}^n{y}_i}{n} $$

where *y*_*i*_ denotes the recorded number of rabies cases in area *i*, *n* is the number of areas, and *W* is the so-called spatial weights matrix, whose element *W*_*ij*_ records the spatial relation between area *i* and area *j*. When the spatial relations are described by a binary matrix, the element *W*_*ij*_ is set to one whenever area *i* is a neighbor of area j, and zero otherwise. If the I statistic is greater than 0, the spatial relationship exhibits positive correlation; correlation is negative when I is negative. The larger the I statistic, the higher the correlation is; in other words, a region close to regions of observed rabies cases is more likely to experience rabies outbreaks, and vice versa.

### Model specifications

When dealing with panel data collected over a large territory, the variance among different sub-regions cannot be ignored. Regions far away from each other can have drastically different socioeconomic or physical environments and consequently fall into different patterns. The effects originating from different patterns can hardly be fully narrated only by explanatory variables. Additionally, components of the effects may derive from some unknown or incidental factors so that the effects turn to be arbitrary. To incorporate spatial heterogeneity and control its unobserved shifting effects, the equation is enhanced with variable intercepts:

$$ {R}_{it}={\beta}^T{X}_{it}+{\alpha}_i+{\eta}_i,{\eta}_i\sim N\left(0,{\sigma_i}^2\right) $$

where *R*_*it*_ is the normalization numbers of rabies cases for area *i* at time *t*; *X*_*it*_ is the vector of predictors; *α*_*i*_ denotes a corresponding intercept for area *i*, which implies a specific level of disease incidence for this area, and thus reflects the spatial heterogeneity of rabies’s impact factors. *η*_*i*_ represents the factors specific to region *i* that are not taken into account by the observed and intrinsic independent variables of rabies’ onset; *η*_*i*_ is a Gaussian random variable with zero mean and controlled by variance *σ*_*i*_^2^.

Spatial dependence is handled explicitly in our analysis. Spatial autocorrelation measures the dependence between geographic objects, which is often depicted as a spatial dependence or spillover effect. Positive autocorrelation indicates that variable values similarity appears in the neighborhood, whereas negative autocorrelation involves considerable discrepancy in values assumed by nearby areas. Positive correlation among disease incidences in adjacent areas can be anticipated. Indeed, adjacent areas often share environmental conditions. With similar natural factors such as humidity, temperature, and slope, the rabies virus and animal vectors tend to have analogous survival and diffusion capacities. As a compounding consideration, similar socioeconomic factors such as traffic conditions, household incomes, educational attainments, and public health policies bring out similar post-exposure handling situations, while the proximity of areas also translates into more regular and intensive communication, such as the periodic movement of the animals that carry the virus.

To measure these neighborhood-based effects, we assume that the count of cases follows a spatial autoregressive process. Both substantive and error spatial dependences are concerned [33]. A Spatial Lag Model (SLM) is suitable for spatial substantive dependence,which is the interaction between the dependent variable and explanatory variables in their geographic vicinity through spillover across region boundaries. A Spatial Error Model (SEM) is suitable to handle spatial error dependence, which is also known as the spatial autocorrelation model; this model can simulate the spatial dissemination of random effects from factors that are not covered by the set of explanatory variables.

Then we attempt to treat both spatial effects simultaneous in a unified model. Therefore, a model that considers spatial dependence both in substantive attributes and in errors is a combination of SLM and SEM. This model consists of a complete SLM model supplemented by a component of simulated spatial random effect from the SEM model, as follows [34]:

$$ R={\beta}^TX+{I}_T\bigotimes \upalpha +\left({I}_T\bigotimes {\rho}_l{W}_N\right)R+u $$

with *β*^*T*^*X* + *I*_*T*_ ⨂ α + η + (*I*_*T*_ ⨂ *ρ*_*l*_*W*_*N*_) as the expression of the SLM. The SLM, also known as the spatial autoregressive model, is employed to represent a system comprised of *N* areas and *T* time periods. In this model, ⨂ denotes the Kronecker product; R is an *NT* × 1 space-time panel matrix of stored and normalized rabies cases; *X* is a *NT* × *k* matrix of explanatory variables; *I*_*T*_ is a time matrix that reflects the changes in the level of rabies outbreak over time; α is a *N* × 1 vector of intercepts identifying the unobserved regression factors of rabies at various periods, whose value is not related to spatial relationships. Also, *ρ*_*l*_ is the scalar parameter for the autoregressive process and *W*_*N*_ is a *N* × *N* spatial weights matrix. In our work, a binary matrix is used and each element [*W*_*N*_]_*ij*_ = 1 signifies that regions i and j are adjacent; otherwise, it is 0. The diagonal elements of *W*_*N*_ are set to zero, and the scalar *ρ*_*l*_ is limited to |*ρ*_*l*_| < 1; hence *I*_*N*_ − *ρ*_*l*_*W*_*N*_ is nonsingular.

In our model, *u* is associated with the SEM model and simulates the spatial random spillover effect. This model component cannot be expressed as a function of the explanatory variables. It is given by:

$$ u=\left({I}_T\bigotimes {\rho}_e{W}_N\right)u+v $$

where *u* is a *NT* × 1 space-time panel matrix of error terms that follow a spatial autoregressive process; *ρ*_*e*_ is the scalar parameter for this autocorrelation process and |*ρ*_*e*_| < 1; *I*_*N*_ − *ρ*_*e*_*W*_*N*_ is also nonsingular. The disturbance term *v* is expressed as follows:

$$ v=\left({e}_T\bigotimes {I}_N\right){\mu}_N+{\vartheta}_N $$

where *e*_*T*_ is a *T* × 1 vector of ones. It encompasses two error terms. *μ*_*N*_ is a *N* × 1 vector of unit specific error components, while *ϑ*_*N*_ is a *TN* × 1 vector that contains error components that vary over areas and time periods. *μ*_*N*_ and *ϑ*_*N*_ are random vectors with zero means and their covariance matrices are $E\left({\mu}_N{\mu_N}^T\right)={\sigma_{\mu_N}}^2{I}_N$ and $E\left({\vartheta}_N{\vartheta_N}^T\right)={\sigma_{\vartheta_N}}^2{I}_{TN}$. Adopting the assumptions proposed by Kapoor et al. [31], the elements of *μ*_*N*_ are identically distributed and so are the elements of *ϑ*_*N*_.

### Estimation approach

We resort to several possible estimators to provide comparisons between classical regression models and models that allow for spatial heterogeneity and spatial dependence effects.

The estimation starts with a baseline OLS estimator derived from pooled OLS equations and ignoring spatial heterogeneity and spatial autocorrelation. The OLS model concentrates on measuring the associations between the dependent variable and explanatory variables.

Second, a random effects-generalized least squares (RE-GLS) estimator is used to represent the gains brought out by spatial heterogeneity. The RE-GLS estimator does not consider spatial dependence.

In order to identify gains derived from spatial effects, a SLM estimator and a SEM estimator must be exploited. Given the presence of spatial autocorrelation, the least-squares estimator is biased and the General Method of Moments (GMM) is employed as an estimation tool to obtain consistent estimates for unknown parameters. The estimators for SLM and SEM here do not consider spatial heterogeneity.

Finally, with heterogeneity and spatial effects both allowed for, an estimator for the aggregation model is built using GMM. The estimator is defined on the basis of the BP-FGLS system estimator proposed by Baltagi and Pirotte [35]. The estimated parameters are obtained through an iteration of two steps. In the first step, $\overset{\sim }{\beta }$ and $\overset{\sim }{\upalpha}$, which denote parameters corresponding to explanatory variables and intercepts, respectively, are calculated using a feasible general least-squares (FGLS) method. An OLS estimator can supply the initial parameters. In the second step, with $\overset{\sim }{\beta }$ and $\overset{\sim }{\upalpha}$ fixed, ${\overset{\sim }{\rho}}_l$ and ${\overset{\sim }{\rho}}_e$ can be estimated by GM estimators proposed by Kapoor. Several different moments are adopted to output unbiased estimators for the scalars. Furthermore, residuals can be calculated with the parameters fixed, and then the variance matrices are estimated using the residuals. The estimated variance matrices are used in the FGLS estimation in the first step in the next iteration. When the estimator converges, we can get an approximate result for the required parameters.

### Comparison of model performances

To estimate the goodness of fit (GOF) of models, the sum of squared residuals and the standard error of regression are used as indicators of model performance. Generally, GOF of regression models can be measured by classical statistics like R-squared or F-statistic. However, the classical statistics are not suitable for GMM estimators. In order to verify the validity of models using GMM, J-statistic and corresponding probabilities are adopted. To provide a general measurement, the sum of squared residuals and the standard error serve as the reference. Furthermore, scatterplots of observations against predictions and residual graphs are illustrated to describe the regression results.

### Data presentation

#### Human rabies data

The data on human rabies cases used in this study are from the National Notifiable Disease Reporting System (NDRS), the national information system of infectious disease mandatory notification of mainland China. Human rabies is classified as a B notifiable disease by the Law of the People’s Republic of China on Prevention and Treatment of Infectious Diseases. All related information is provided by NDRS.

The data set contains 20,368 reported cases of human rabies, spanning from 2005 to 2013. The cases pertain to humans infected by dogs, cats, rats, bats and other transmitters. Among them, dogs are believed to be the most important transmitter and cause over 80 % of cases of human rabies in China. Although the detailed proportion of all transmitters of human rabies cases was not available for this study because of the insufficiency of the information (demographic and clinical data) for part of the cases, we can acquire information on the virus transmitters through analyses of a sample of all cases. Analysis of human rabies cases in Guangdong province in 2003 and 2004 suggested that 85.7% of all cases were infected by dogs, followed by cats (3.7%) and rats (2.5%) [36].

Each suspected human case would be mandatorily reported to public health authorities. All reported cases with geocodes (i.e. longitude and latitude of the household home address) were included in our study. Four suspicious cases (found in 2005, 2008, 2011 and 2011, respectively) in Xinjiang Province were dropped from our analysis. The China Centers for Disease Control (CDC) ethical committee approved our research on these data and the data were anonymized.

**Fig. 1:**
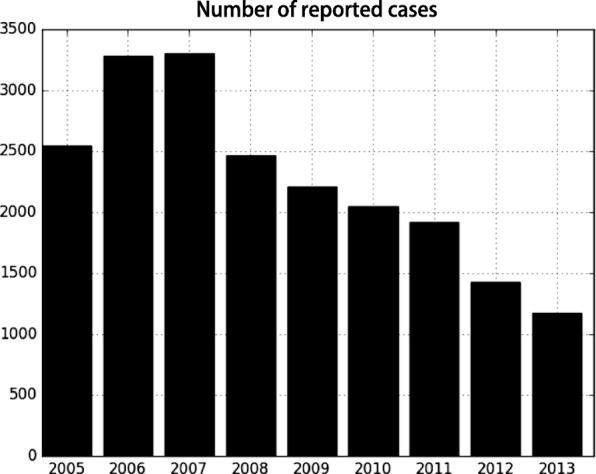
The numbers of reported rabies cases from 2005 to 2013 [40]

**Fig. 2:**
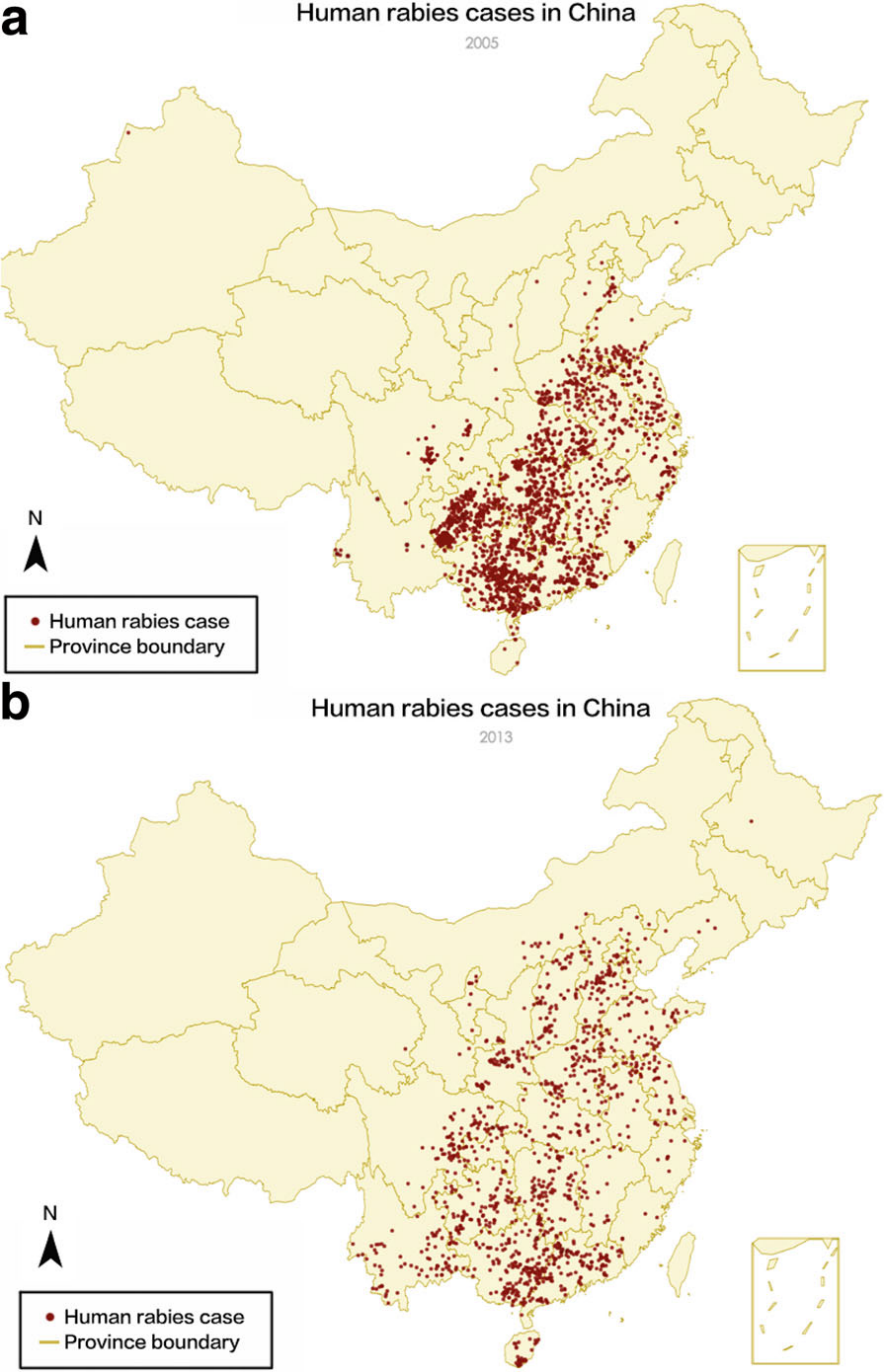
Maps of human rabies cases in China in 2005 (a) and 2013 (b). The map encompasses 23 provinces, 5 autonomous regions and 4 municipalities under the direct control of the central government

Figure 1 [40] displays the numbers of rabies notification cases from 2005 to 2013. A downward trend of the count of cases, which first manifested itself in 2008, is depicted in the figure. However, the annual number of cases has remained high and the corresponding burden cannot be simply ignored. Therefore, it is fitting to seek to identify the factors that led to the downward trend of rabies cases in China during this nine-year timespan. Figure 2 represents the spatial distribution of human rabies cases in China. According to these maps, cases are more frequent in the eastern and southern parts of China, with a spatial pattern that has shifted over time.

For statistical analysis, each case was assigned to an administrative village, which served as the primary sampling unit. The number of administrative villages in China is so large that the result of this assignment turned out to be extremely sparse. Therefore, the primary unit for the regression analysis was set to be a cell instead of an administrative village. The cells were obtained through spatial clustering: villages that are adjacent or have direct geographical connections with each other and share the same or similar environmental conditions, were aggregated. Each cell consisted of several villages. The number of villages contained in a cell and the spatial extent of a cell vary according to the size and condition of relevant administrative villages. Some rivers and (mountain) watersheds are not only natural unit boundaries but they are also the administrative boundaries, so there is a greater possibility that our cell boundaries overlap with the administrative boundaries in these instances. Due to the variability in the geographic characteristics of the statistical units that are grouped into the same cell, aggregation errors may be generated. However, because the aggregation was conducted so as to minimize information loss, aggregation errors should be small and rather uniform, thus mitigation impacts on the national scale analysis. Through this process, we obtained 271 spatially defined cells for the subsequent regression analysis.

#### Environmental and socioeconomic data

Explanatory variables were selected from a set of features that denote the geographic, climatic and socioeconomic conditions of a specific region. The environmental and geographic features included: the longitude and the latitude, the average annual land surface temperature (LST), the slope and the average elevation. To get the average value for a specific year, an adaptive Savitzky-Golay smoothing filter, implemented using the TIMESAT package [37], was employed. The socioeconomic features pertained to the human population density, yearly gross domestic product (GDP), per capita GDP, ratio of middle school graduates (RMS), and ratio of illiteracy (ROI). Data on human population were obtained from three national censuses (2000, 2005 and 2010) released by the National Statistics Bureau of China. The values of population for intervening years were estimated using a linear interpolation method. Furthermore, a spatial trend analysis methodology [38] was used to smooth data on several neighboring villages and provide equivalent values for the corresponding cell.

Also, the set of covariates was extended with transportation and spatial accessibility features, which included the Euclidean distances from a village to the road network, to the nearest downtown of a county or a city, to the nearest hospital, and to the nearest clinic. The smallest offset to the road network was used to describe the distance to the road network. Public health measures, including various programs for the prevention and control of the disease, are in effect restricted by the local traffic and accessibility conditions.

Finally, spatial epidemiology variables were incorporated. The epidemiologic features included the minimum spatial distance to the nearest case, the minimum temporal distance to the latest case, and the minimum spatio-temporal distance to the nearest case. Epidemiology variables may reveal correlations between infected zones. Moreover, the distance from nearest or latest case represented the degree of potential risk revealed by existing cases. Explicit information on the full set of variables is listed in Table 1.

**Table 1:** List of explanatory variables

Category	Description of dataset	Abbreviation	Unit	Data source
Environmental variables	digital elevation	DEM	m	USGS
	digital slope	SLOPE	degree	USGS
	Average temperature	AT	^°^C	MODIS
	Human population density 2000	POPDENS	p/km^2^	National Statistics Bureau
Socioeconomic variables	Human population density 2005	POPDENS	p/km^2^	National Statistics Bureau
	Human population density 2010	POPDENS	p/km^2^	National Statistics Bureau
	Ratio of illiteracy	ROI	p/million	National Statistics Bureau of China
	Ratio of middle school and above	RMS	p/million	National Statistics Bureau of China
	Yearly GDP	GDP	10^4^RMB	National Statistics Bureau of China
	Yearly per Capita GDP	PCGDP	10^4^RMB	National Statistics Bureau of China
	Distance to road network	DTRN	km	National Administration of Surveying, Mapping and Geoinformation
Transportation variables	Distance to city center	DTCC	km	National Administration of Surveying, Mapping and Geoinformation
	Distance to county center	DTCNC	km	National Administration of Surveying, Mapping and Geoinformation
	Distance to nearest hospital	DTHSP	km	China’s Health and Family Planning Commission
Epidemiologic variables	Distance to nearest clinic	DTCLC	km	China’s Health and Family Planning Commission
	Minimum spatio-temporal distance to nearest case	MSTDNC	Km/day	China CDC Rabies Surveillance data
	Minimum spatial distance to nearest case	MSDNC	km	China CDC Rabies Surveillance data
	Minimum temporal distance to nearest case	MTDNC	day	China CDC Rabies Surveillance data

#### Variable selection

Multiple backward stepwise regressions were carried out to select meaningful explanatory variables. The stepwise process was repeated 1000 times applying different training subsets. The 20 regression models with the best fit were picked, while the variables yielding non-significant effects (mean *P*-value > 0.05) were removed.

At the end of this process, the variables that were retained included the longitude, the average temperature, the distance to county center, the distance to road network and the minimum spatial distance to the nearest case. More detailed results are presented hereunder for this specification.

## Modeling results

### Spatial autocorrelation

**Fig. 3:**
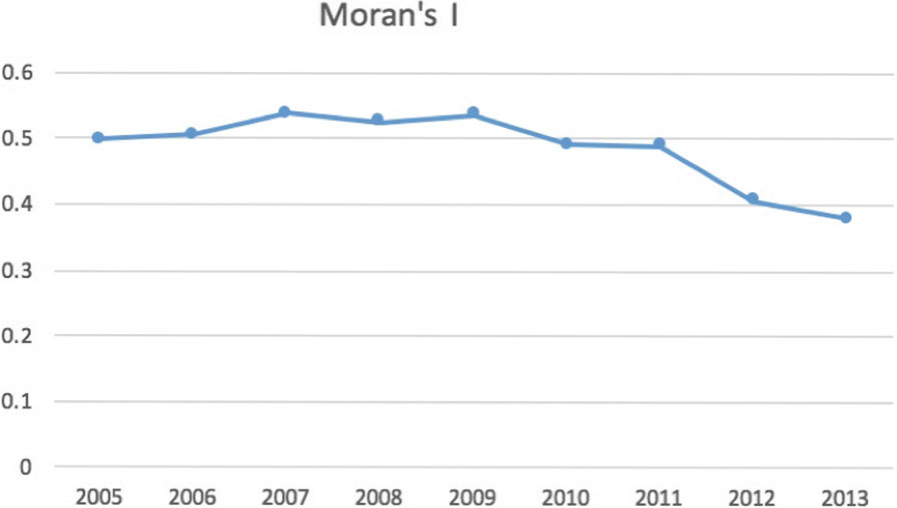
The Moran’s I index, 2005 to 2013 [40]

The Moran’s I computed on the normalized case count for 2005 to 2013 is illustrated in Fig. 3 [40]. The global index is statistically significant and positive; it provides evidence of positive spatial autocorrelation in rabies incidence in China. The graph also depicts the longitudinal downward trend of Moran’s I, which may be a consequence of the continuous drop of rabies cases in China from 2008.

### Estimation results

The input of the regression models is a balanced panel that contains 271 cross-sections with 9 time periods. Thus, 2439 samples are generated, each of which consists of the normalized rabies incidence and explanatory variables of an individual cell in a specific year. The estimation results are reported in Table 2. AM stands for the aggregation model that allows for both the heterogeneity effect and the spatial dependence effect. The results of the variable selection process show that the variables can be accepted as significant predictors of the normalized number of rabies cases. The J-statistic implies that the SLM, the SEM, and the aggregation model are all valid.

**Table 2:** Estimation results

	OLS		SLM		SEM		RE-GLS		AM	
	Parameter Estimate	Standard Error	Parameter Estimate	Standard Error	Parameter Estimate	Standard Error	Parameter Estimate	Standard Error	Parameter Estimate	Standard Error
Longitude	0.03256	0	0.001901	0.0486	0.002056	0.0331	0.001603	0	0.00222	0
Temperature	0.03812	0.0001	0.011207	0.0004	0.012794	0.0001	0.013616	0	0.007505	0
DTCNC	0.005884	0.0102	0.002337	0.0319	0.002302	0.0360	0.000906	0	0.001898	0
DTRN	0.049686	0.0021	0.011251	0.0093	0.012252	0.0043	0.005268	0.0002	0.006013	0.0005
MSDNC	−0.002092	0.1651	−0.002264	0	−0.002466	0	−0.001311	0	−0.001691	0
C	0.047878	0	0.47645	0	0.455308	0	0.681607	0	0.4073	0.0019
*ρ*_*l*_			0.008774	0					0.005153	0
*ρ*_*e*_					0.117826	0			0.013151	0.0254
${\sigma_{\vartheta_N}}^2$			0.866774		0.874635		1.043152		0.684197	
${\sigma_{\mu_N}}^2$			0.903172		0.876109		0.934437		0.881217	
R-squared	0.3775						0.6691			
J-statistic			74.4461	0	71.3784	0			103.6390	0
sum-resid	5191.23		1311.323		1298.777		2060.687		756.4909	
S.E.	1.461011		0.832959		0.829185		0.976063		0.684197	

Table 2 provides reference for comparisons between different regression models. As is shown in the table, the use of variable intercepts improves the performance of the model by a wide margin. On the other hand, the residual and standard error of regression obviously decrease once spatial autocorrelation is taken into account. The SLM and the SEM provide better performance. The results imply that both the spatial heterogeneity and spatial dependence effects provide a meaningful contribution to understanding rabies risk. When the model combines estimators from SEM and SLM, it reaches the best performance of all models. This may suggest that both spatial substantive dependence and spatial error dependence have a significant influence on the prevalence of rabies cases.

**Fig. 4:**
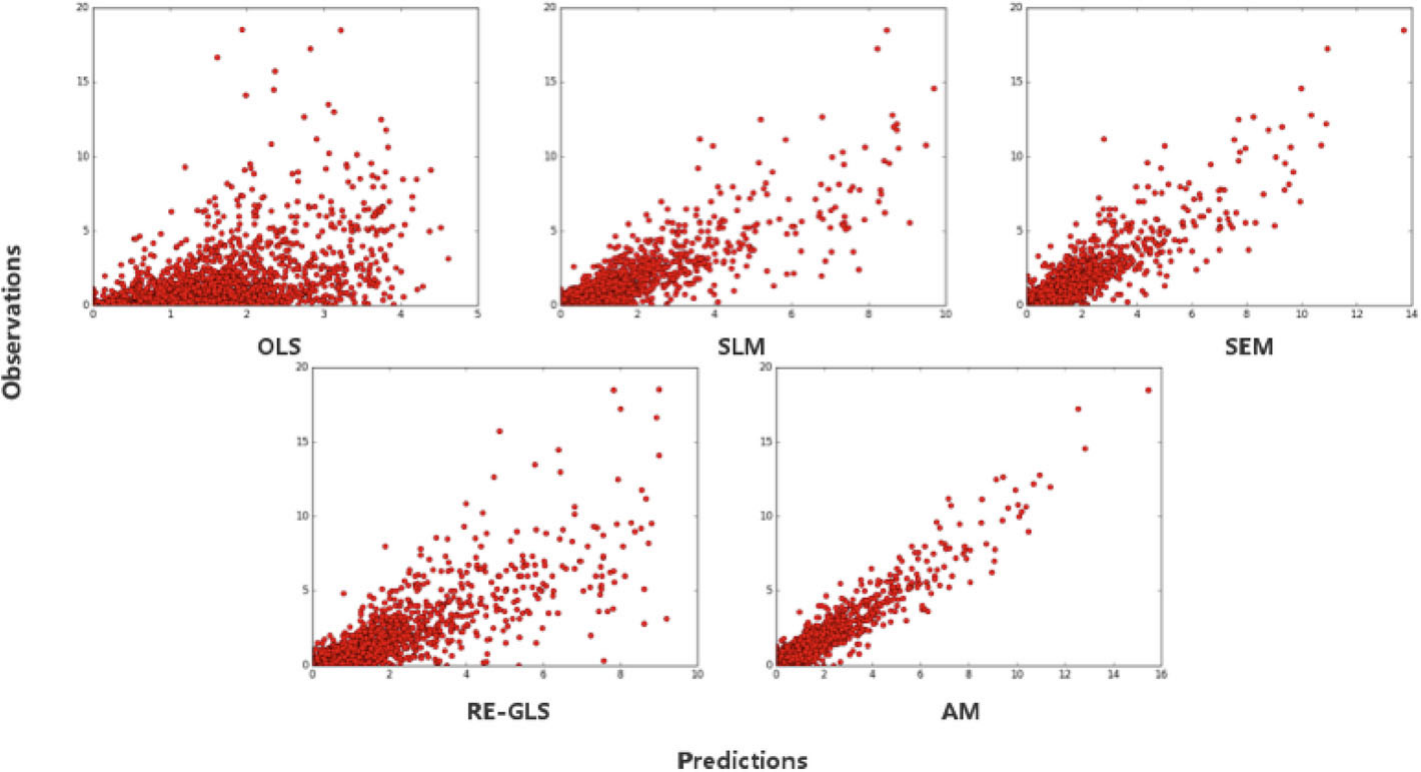
Scatterplots of observed counts (vertical axis) and predicted counts (horizontal axis) of different regression models [40]

Figure 4 shows the scatterplots of observed counts against predicted counts. Each dot denotes a pair of observed and predicted values for any given cell. We find that a preponderance of dots assemble near the diagonal line of the plot when spatial heterogeneity and spatial dependence are explicitly incorporated in the model. The plots prove that measurements for spatial heterogeneity and spatial dependence effects bring effective improvements to the model, which is consistent with the analysis on the result of goodness of fit measures.

**Fig. 5:**
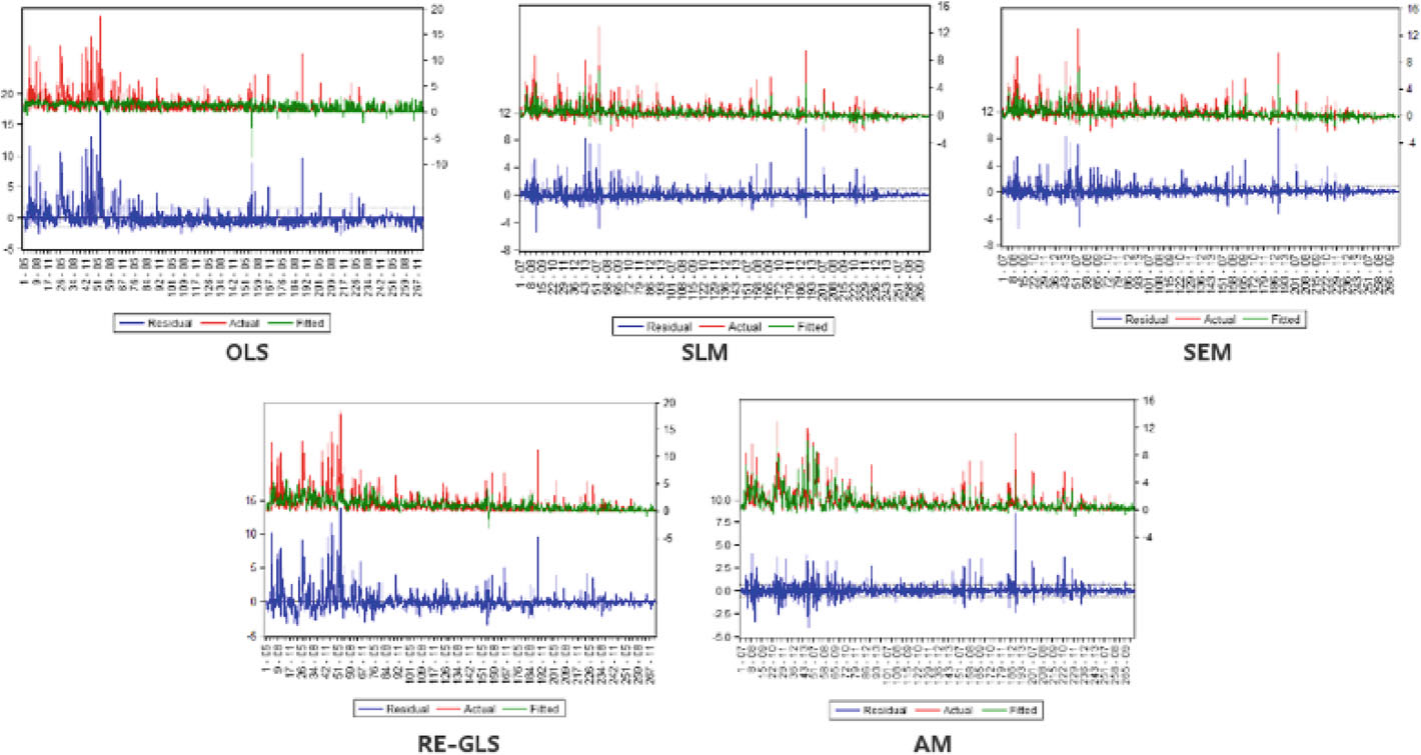
Residual graphs of different models [40]

In Fig. 5, the residual for each sample case is depicted along with their actual and fitted values. The blue lines denote the residuals, while the red lines denote the actual observations. The green lines show the fitted values. The overlap between actual and fitted values grows with spatial heterogeneity and spatial dependence incorporated explicitly in the model. The OLS estimator fails to fit the observations well, especially in the areas with high incidence of rabies. The SLM estimator and the SEM estimator provide improved results, but it is hard to tell the difference between the two spatial dependence processes through the residual graphs. The RE-GLS estimator also gives better performance than the OLS estimator. The graphs suggest that accounting for spatial dependence leads to more improvements than spatial heterogeneity in this case. Furthermore, when spatial heterogeneity and spatial dependence effects are both considered, the aggregation model provides the best fit to the observations.

**Fig. 6:**
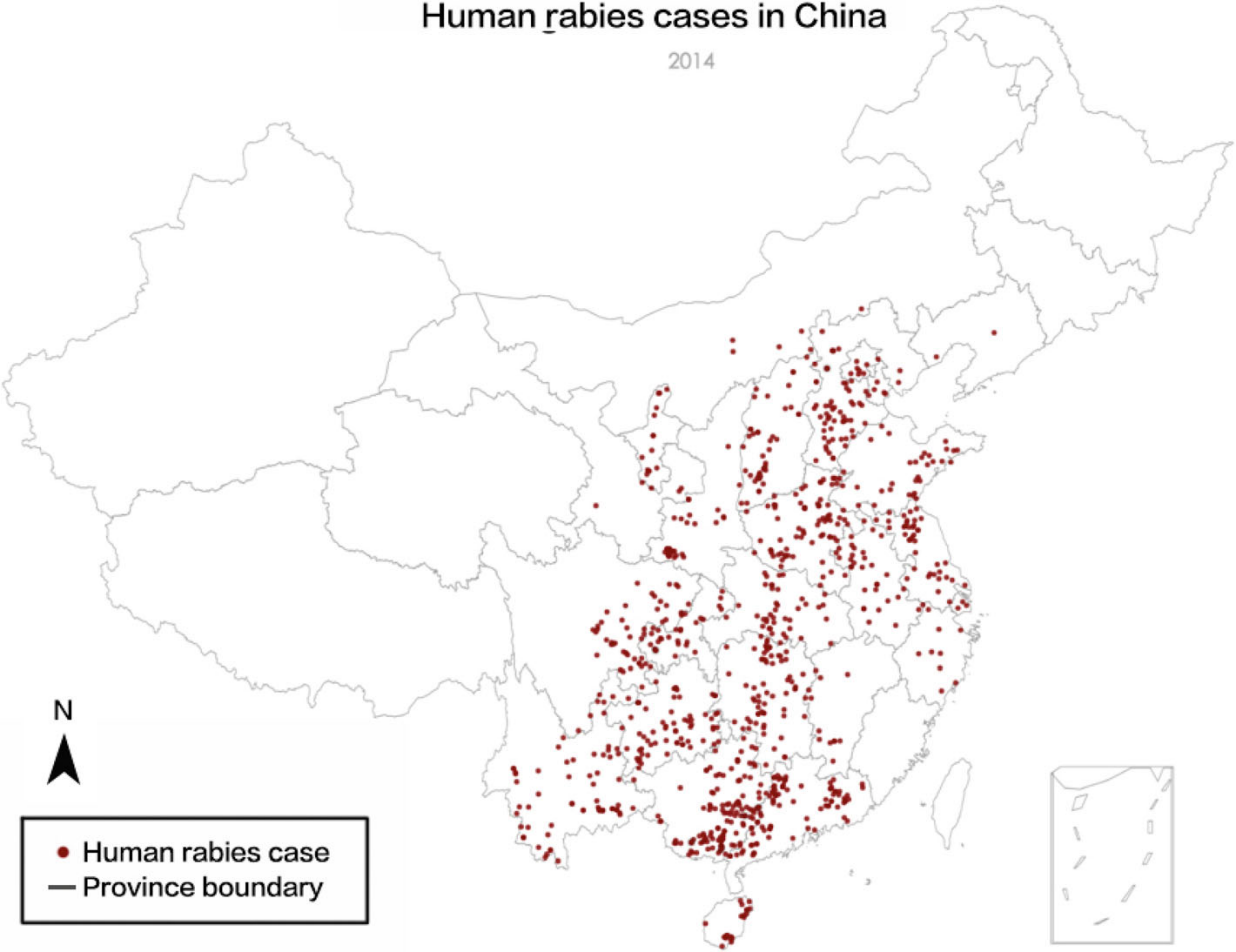
Map of reported rabies cases in 2014

**Fig. 7:**
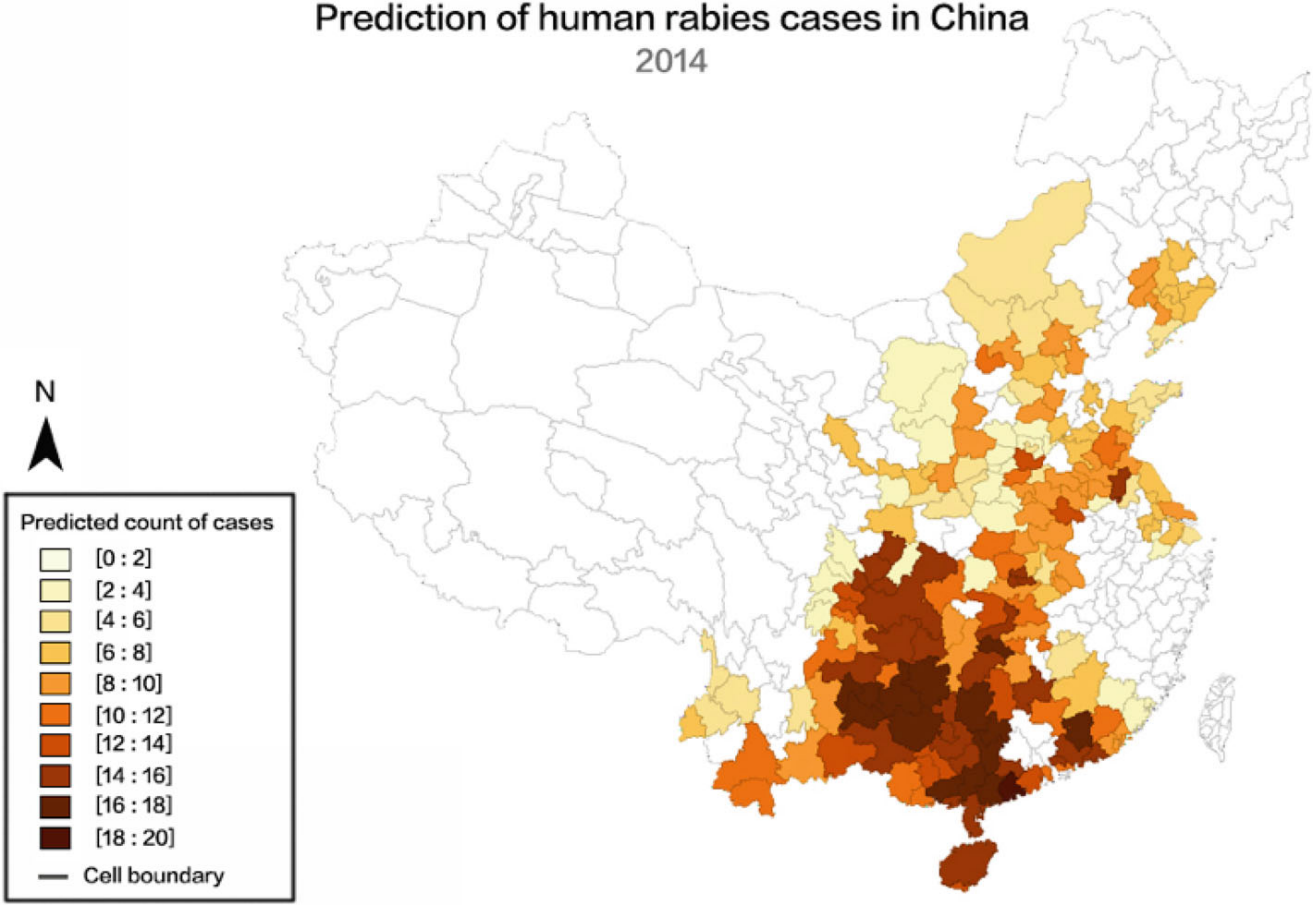
Map of predicted counts of cases in 2014

Figure 6 shows the distribution of actual human rabies cases in 2014 while Fig. 7 shows the prediction of human rabies cases in the same year according to the AM. The color depth in Fig. 7 denotes the possible extent of the disease; darker colors suggest more anticipated cases in the corresponding regions. Generally speaking, in comparison with the map of observed cases in 2014, the model simulation is good. However, the accuracy of simulation in the two regions of eastern Anhui and Zhejiang Provinces is relatively low. This may be due to the following reasons: (1) rabies in Anhui is mainly concentrated in the northwestern part of the province; (2) Zhejiang and Anhui Provinces are not areas of high incidence of rabies. The two provinces have flat terrain, and the road traffic connectivity is relatively good, so that rabies simulation accuracy is relatively low. It can be concluded that the prediction fits the observations well in most regions of China.

## Discussion

In section “Discussion”, we compared the performance of different regression models and verified the validity of the models that incorporated spatial heterogeneity and spatial dependence effects. In this section an interpretation of the estimates is provided.

According to the *P*-values in Table 2, the selected variables can be accepted as effective independent variables for the regression of normalized rabies counts. The longitude and the average temperature both show positive correlation with the count of rabies. The longitude of an area is significant in the regression, as it reflects the environmental attributes across China on a relatively macro level. Physiographically, China can be separated into three divisions: the eastern part with low altitude and a long coastline; the middle part with numerous mountains and basins; the western part with high altitude and plateaus. The climatic environment varies according to the longitude and the differences between the eastern part and the western part can be enormous. Generally, from west to east, as the longitude increases, rainfall and vegetation tend to be relatively higher, and the environment is more suitable for the survival and reproduction of animals, including rabies virus carriers. Moreover, the middle-western part consists of many plateaus and rugged mountainous regions, which is not fit for animals, like stray dogs, to roam. In contrast, in the plain areas, if the distance separating villages is larger than the range of roaming dogs, rabies can hardly spread through stray dogs. This suggests that different prevention and control measurements should be adopted in mountain regions and plain regions. For mountain regions, the key means is to control the movement and propagation of stray dogs inside the area, especially in rugged and forested landscapes. In the plains, however, the target is to control the connectivity between the infected villages, where human rabies cases have been recorded, and un-infected villages.

The incidence of rabies also increases as ambient temperature rises. A warmer climate means that animals are more active in their surroundings and track over greater distances, which contributes to the spread of rabies. In addition, higher temperatures often result in humans wearing lighter clothes and in exposing more skin, which increase the opportunity of being bitten by dogs.

Table 2 also suggests that the incidence is affected by transportation accessibility and spatial epidemiology considerations. The Euclidean distance to the road network and the distance to the nearest county center have positive coefficients in the regression. Distances can reflect the traffic conditions of an area and the intensity of connections with socioeconomic resources. The longer the distance is, the more isolated a region may be with respect to other communities. As a result of this isolation, the area receives less support for disease prevention and other public health interventions. Moreover, restricted by the limitations of local resources, the area would only have a slower response to rabies cases and thus be even more afflicted. According to WHO guidelines for post-exposure prophylaxis [39], it is critical to receive PEP in a rabies center as soon as possible after exposure. Although 98% of all patients were living in rural areas, great differences were recorded in the speed of seeking medical assistance: 64.19% of patients visited the rabies center within 4 h of exposure, 27.93% of patients visited the rabies center between 4 and 24 h of exposure and 7.88% of patients visited the rabies center over 1 day later [24]. These differences may be largely imputed to accessibility discrepancies. Although we did not collect information on PEP hospitals and rabies centers, the accessibility to these facilities can be revealed by the distances mentioned above. The distance to the nearest county center translates into the convenience of access to receive rabies PEP and immunoglobulin services.

The count of rabies has a negative correlation with the spatial distance to the nearest case. A negative coefficient implies that when the distance from existing cases becomes shorter, the risk of infection rises correspondingly. When one human case occurs, the rabies virus has spread among hosts in the regions near this case. Strict control and protection measures should be adopted in these regions. In current human rabies control and prevention plans issued by the Ministry of Agriculture of China, control areas include two buffer areas centered on the location of rabies cases, namely the infected areas (within a radius of 3 km) and the risk areas (within a radius of 5 km, excluding the infected areas). In the infected areas, the local CDC and the government will cull infected dogs and restrict others’ movements. To control the rabies transmission, mandatory vaccination of dogs should be enforced in both the infected areas and the risk areas.

patial heterogeneity has been demonstrated to contribute to the improvement of the fit of the regression. The RE-GLS estimator provided a base constant and each area was fitted with an adjusted constant as its intercept. The incorporation of spatial heterogeneity greatly reduced the residuals of the regression. With spatial dependence accounted for, the constant was updated to fit the new model. The SLM and SEM also led to a further drop in the residuals. The corresponding *P*-values of *ρ*_*l*_ and *ρ*_*e*_ have proven the significance of spatial heterogeneity and spatial dependence effects. The results showed that the variance of the random effects (${\sigma_{\vartheta_N}}^2$ and ${\sigma_{\mu_N}}^2$) are also significant at 1% for all models. Furthermore, the aggregation model evidenced the successful combination of the two factors. The spatial heterogeneity and dependence effects are both actually recognized to play important roles in the spread of the disease in China. Consideration for differences across areas and for interactions between areas suggests the wisdom of some degree of local and decentralized decision making on the part of government agencies and medical institutions. As discussed above, effective strategies for controlling the disease are supposed to fit the specific conditions of the socioeconomic and physical environments in the localities. The adjacency relationships between localities need extra attention, for it may reveal the spread source of rabies in neighboring areas and it can be exploited to interrupt the path of the transmission.

## Conclusions

In this research, we analyze human rabies in China using regression models with consideration for spatial heterogeneity and spatial dependence effects. We studied rabies cases recorded in China from 2005 to 2013 and applied regression models based on normalized case data. The regression estimates provide a reference for measuring effects concluded from explanatory variables whose significance were then extracted including the longitude, the average temperature, the distance to town center, the distance to the road network and the spatial distance to the nearest rabies case. The analysis explained inferred relationships between the case counts and relevant explanatory variables. For instance, the survival chance of stray dogs is higher when temperature is higher and less clothes are worn to protect from biting dogs. In rural areas, longer average distances between villages and town centers and greater distance to road network mean that it is more difficult to have timely PEP treatment. Given the variables identified as strong predictors, recommendations on how to prevent and control human rabies were presented.

Moreover, spatial heterogeneity and spatial dependence were explicitly considered in our analysis. Spatial autocorrelation is confirmed by Moran’s I statistic. For a specific area, the incidence of rabies is affected by both its constant and inherent attributes, and the status of neighboring areas. The omission of the heterogeneity and spatial dependence effects can bring biased estimations, which has been proven by the results of pooled OLS estimation. Through comparisons between traditional models and the aggregation model that allows for the two types of effects simultaneously, we demonstrated the validity and advantage of the aggregation model. The aggregation model outperformed the existing models and fitted the observations well. The comparisons suggested that both spatial heterogeneity and spatial dependence effects can contribute to the model and that they can be combined in a single model without any interferences, thus effectively reducing the possibility of bias.

However, challenges remain for the spatial epidemiologic modeling of rabies. Although our approach obtained promising results on the dataset of rabies cases in China, it still needs to be verified on other datasets. While the approach that is advocated here recognizes the role of heterogeneity to reveal new insights in terms of missing variables and inherent characteristics of different regions, the aggregation of areas leads to the loss of fine-resolution information on villages assigned to a larger cell. Therefore, how to better account for the conditions within a cell and how to map the individual effects may be interesting issues to tackle in future extensions of this work. In addition, in this study, the time increment was set to a year and seasonal effects were not taken into consideration. Alternatively, it is possible to compute the monthly count of rabies cases and conduct relevant analysis according to the rhythm of seasons. In future work, we will enhance the specification of the aggregation model and test it on various datasets.

## Abbreviations

AM: The aggregation model that allows for both the heterogeneity effect and the spatial autocorrelation effect
CDC: Centers for disease control
FGLS: Feasible general least-squares
GDP: Gross domestic product
GMM: General method of moments
GOF: Goodness of fit
LST: Land surface temperature
MODIS: Moderate Resolution Imaging Spectroradiometer
NDRS: Notifiable disease reporting system
OLS: Ordinary least squares
PEP: Post-exposure prophylaxis
RE-GLS: Random effects-generalized least squares
RMS: Ratio of middle school graduates
ROI: Ratio of illiteracy
SEM: Spatial error model
SLM: Spatial lag model
SUR: Seemingly unrelated regression
TIMESAT: A program package for extracting seasonal parameters from remotely sensed time-series data of vegetation
USGS: United States Geological Survey
